# Inter- and Intra-Individual Variation in the Behavior of Feed Intake on Nutrient Availability in Early Lactating Dairy Cows

**DOI:** 10.3390/ani12010037

**Published:** 2021-12-24

**Authors:** Theresa Rumphorst, Theresa Scheu, Christian Koch, Albert Sundrum

**Affiliations:** 1 Department of Animal Nutrition and Animal Health, Faculty of Organic Agricultural Sciences, University of Kassel, Nordbahnhofstr. 1a, 37213 Witzenhausen, Germany; 2 Educational and Research Centre for Animal Husbandry, Hofgut Neumuehle, 67728 Muenchweiler an der Alsenz, Germany; t.scheu@neumuehle.bv-pfalz.de (T.S.); c.koch@neumuehle.bv-pfalz.de (C.K.)

**Keywords:** dairy cows, variation, digestibility, dry matter intake, intake behavior, nutrient availability

## Abstract

**Simple Summary:**

The problem of nutrient and energy deficiency and the associated risk of disease in fresh dairy cows has been known for many years. Previous approaches to reducing the risk have been almost exclusively on a herd basis approach but have so far not been sufficiently effective. The present study revealed large variation between individual animals during the first weeks after calving, particularly with respect to dry matter intake (DMI). In addition to the large variation in intake behavior, feed intake and nutrient digestibility, interactions between parameters refute the traditional feeding regimes, based on the mean requirement values at herd level. Inter-individual variation indicates that each animal follows an individual strategy in optimizing DMI. Only if constant access to the feed bunk and balanced diets are made possible, all animals can follow their individual strategy, maximize individual DMI and come closer to the goal of an adequate supply. As there is only a low risk for excessive DMI during early lactation, feeding regimes should not be oriented towards the assumed average level of feed intake but towards the animals with a low level of dry matter intake. Otherwise, it will not be possible to improve the nutrient supply for all animals.

**Abstract:**

Since energetic deficits in dairy cows can only be reduced at an animal level, the objective of the present study was to determine the extent of variation in intake behavior within and between animals during early lactation, to explore the magnitude of interactions between feed intake, intake behavior and nutrient digestibility, and to identify levers for maximizing feed intake at the individual animal level. Feeding behavior, intake and nutrient digestibility of 28 German Holstein dairy cows, fed TMR with 7.0 MJ NEL, were studied between the 2nd and 15th week after calving. Dry matter intake was assessed daily and nutrient digestibility weekly, with iNDF_240_ as an intrinsic marker. Results showed high intra- and inter-individual variation in intake behavior parameters with coefficients of variation (CV) up to 0.58 in meal frequency. Nutrient digestibility varied only slightly with CV values up to 0.10 in crude protein. Milk yield, meal frequency, feeding time, feeding rate and meal size had significant positive effects on DMI (*p* < 0.01). To achieve long-term improvements in feed intake, it is important to optimize feed intake and feeding behavior of individual animals by improving feeding conditions and develop technical tools to identify animals with insufficient feed intake.

## 1. Introduction

**Figure 1:**
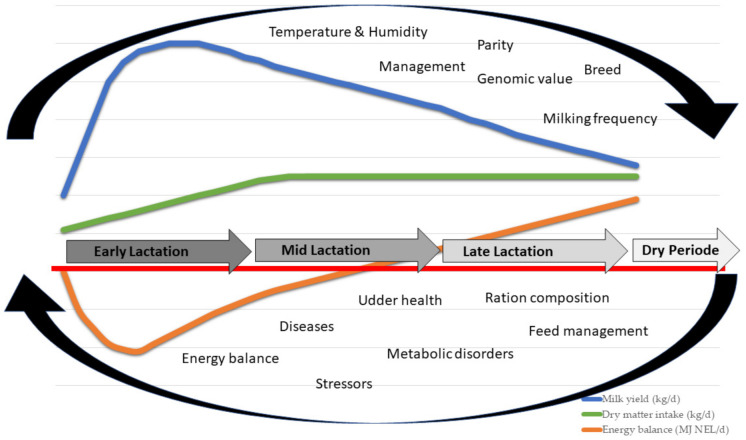
Schematic representation of factors affecting milk yield, dry matter intake and energy balance of dairy cows during different states of lactation [6].

Many high-yielding dairy cows suffer severe nutrient and energy deficits in early lactation [1,2]. These deficient states stress body functions and reduce an animal’s resistance to stressors while increasing the risk of metabolic and infectious diseases [3,4,5]. Especially in early lactation, many factors affect the performance and health status of dairy cows, but the level of influence can vary between individual animals (Figure 1).

The magnitude of nutrient and energy deficiencies also varies between dairy cows and depends on nutrient and energy intake and requirements. In addition, there is a large variation between individual cows in their ability to cope with metabolic imbalance [2]. The individual availability of nutrients and energy results from the combination of feed components in the diet, the level of feed intake and the degree of digestibility of the feed in an animal, as well as the variation and interaction within and between these factors. Of these factors, feed intake has proportionately the greatest impact on the level of nutrient and energy availability. Accordingly, research in recent years has focused on optimizing diet composition in relation to the average requirements of the dairy cows in the feeding group to achieve a high level of dry matter intake (DMI) [7,8,9]. While feeding behavior showed a considerable effect on the level of DMI [10,11], it is often not adequately considered in ration calculation, feeding management or nutrient supply. In addition, it is difficult to assess feeding behavior in practice. Feeding behavior can be described by the number, duration and size of individual meals per day [12]. DMI, as the sum of single meals, is influenced both positively and negatively by the individual behavior patterns as well as their interactions [10]. Furthermore, an influence on the digestibility of the ingested diet has also been described. Baumont et al. [13] and Golden et al. [14] revealed that cattle and small ruminants which ate smaller and more frequent meals showed higher nutrient digestibility than animals that ate less frequent, but larger, meals. Tyrrell and Moe [15] and Potts et al. [16] found that digestibility diminished with increasing feed intake. Higher feed intake results in a higher passage rate, which leads to a reduced digestion time. However, the extent of variation among individual animals and the degree of antagonistic relationships between feeding behavior, feed intake and digestibility have so far received little attention. Since individual deficiencies can only be reduced at the animal level, the objective of the present study was to determine the extent to which intake behavior and nutrient digestibility varies within and between animals during early lactation, to explore the extent of interactions between feed intake, intake behavior and nutrient digestibility, and to identify levers for maximizing feed intake and hence nutrient intake at an individual animal level.

## 2. Materials and Methods

All procedures described in this study were performed according to the German Animal Welfare Act and approved by the local authority for animal welfare affairs (Landesuntersuchungsamt Rheinland-Pfalz; G 18-20-073) in Koblenz, Germany.

### 2.1. Animal Housing and Diet

The study was carried out between April and October 2018 at the Educational and Research Centre for Animal Husbandry, Hofgut Neumuehle, Muenchweiler a.d. Alsenz, Germany. A total of 28 German Holstein dairy cows ranging from the 2nd to 8th parity (mean = 2.9; SD = 1.3) and between the 2nd and 15th week of lactation were used. German Holsteins are the most common dairy breed in Germany and play a crucial role in the German dairy industry. Parity and mean daily milk yield (kg/d) of every single cow during the course of study were shown in Table 1.

The cows were housed together with non-experimental cows in a free-stall barn with 60 cubicles and 30 feeding units. Cows had unlimited access to fresh water and were fed a total mixed ration (TMR) ad libitum (Table 2). The TMR was prepared in the morning and delivered twice daily (60% of total daily amount at 6 a.m. and 40% of total daily amount at 11 a.m.).

### 2.2. Data and Sample Collection

Individual feed intake was measured daily with the Insentec B.V. (Marknesse, the Netherlands) RIC (roughage intake control) automatic weighing system. Cows were identified using individual collar transponders, which registered access to the feeding unit. The connected computer recorded cow number, feeder number, trough weight and the time of the beginning and the end of each visit. TMR intake per visit was calculated from the differences in trough weight between start and end of the visit. Feeding time was calculated from the difference between start and end timepoint. Each visit resulting in a trough weight difference of more than 0.1 kg was considered a meal. All parameters were calculated on a dry matter base by multiplying the amount of fresh matter by the dry matter (DM) content of the TMR. Intake behavior was characterized for an individual animal over a day by the following parameters and definitions, according to Nielsen [12]: meal frequency (meals/d), defined as number of individual feeding bouts per day; meal duration (min/meal), defined as average time per meal, average meal size (kg DM/meal); daily feeding time (min/d), defined as the sum of the meal durations in a day; daily DMI (kg DM/d), defined as the sum of the meal sizes in a day and speed of food ingested; or feeding rate (g DM/min), defined as the ratio between meals size (kg DM/meal) and meal duration (min/meal). To account for the individuality of feed intake behavior, the general term “feeding behavior” is replaced by the term “intake behavior” in all that follows. The individual parameters of daily intake behavior were averaged for every cow per week of lactation. Cows were milked twice daily between 5.00 and 7.30 a.m. and between 3.30 and 6.00 p.m. The daily milk yield was recorded electronically via the herd management system Dairy Plan C21 (GEA Farm Technologies, Boenen, Germany). Milk aliquots from one evening and the next morning were taken biweekly and pooled for further analysis of milk fat, protein and lactose by infrared spectrophotometry using a MilkoScan FT6000 (Foss Analytical A/S, Hillerød, Denmark). TMR samples were taken daily within one hour of feed delivery. Daily samples were combined on a weekly basis with a representative TMR sample of 800–1000 g for determining the weekly dry matter content. Starting at the day of calving, individual cow fecal samples were collected weekly two hours after morning milking via rectal palpation. The samples were labeled and stored frozen at −20 °C until chemical analysis. Deviations in fecal content within a day, especially in aNDFom and iNDF_240_, were reduced by defining a fixed time of feeding and sampling, which remained constant over the test period.

### 2.3. Chemical Analysis

Dry matter content was determined in a two-step process: thawed TMR and feces samples were first oven dried at 60 °C for 48 h and ground to 1 mm particles, followed by drying at 105 °C for 3 h until constant weight was achieved. Organic matter (OM) was measured by ashing (550 °C) overnight. The dried and ground samples were submitted to Cumberland Valley Analytical Services Inc., Waynesboro, PA (CVAS), for chemical analysis. All TMR samples were analyzed for dry matter (DM), organic matter (OM) (method 942.05; [18]), crude protein (CP) (method 990.03; [18]), soluble protein (SP) [19], ether extract (EE) (method 2003.05; [18]), neutral detergent fiber assayed with heat-stable amylase and expressed exclusive of residual ash (aNDFom) [20], acid detergent fiber (ADF), expressed inclusive of residual ash (method 978.10; [18]), lignin (method 973.18; [18]), ethanol-soluble carbohydrates (ESC) [21], starch [22] and 240 h in vitro indigestible neutral detergent fiber (iNDF_240_) [23]. Dried and ground fecal samples were split into two subsamples. One subsample was analyzed with near infrared reflectance spectroscopy (NIRS) described by [24]. The analysis included DM, OM and CP. The other subsample was submitted to CVAS for determination of ADF, aNDFom and iNDF_240_.

### 2.4. Calculations and Statistical Analysis

Sample size was determined by the capacity of the research facility. Our sample size of n = 28 was assumed as adequate to reliably detect differences between cows and weeks of lactation (>0.95 statistical power and *p* ≤ 0.05 significance level; G*power 3 software; [25]). Organic matter digestibility (OMD), crude protein digestibility (CPD), neutral detergent fiber digestibility (NDFD) and acid detergent digestibility (ADFD) were calculated from iNDF_240_ as internal marker and nutrient concentrations in TMR and feces using the following equation, by [26]:

Apparent nutrient digestibility (g/kg) = 1000 − 100 × {[iNDF_240_] Diet/[iNDF_240_] Feces} × {[nutrient] Feces/[nutrient] Diet} (1)

Data was analyzed using the SPSS 25.0 software (IBM Company Inc., Chicago, IL, USA). Each variable was checked for normal distribution by a histogram and a Q–Q plot, and the mean, range, standard deviation (SD) and coefficient of variation (CV) were calculated. Differences were considered significant at a level of *p* ≤ 0.05, and a tendency was considered at 0.05 < *p* ≤ 0.10. Changes of each parameter for all cows over the course of the study are shown by box plots, which represent the median, interquartile range and extreme cases of individual variables. 

The differences in intake behavior and nutrient digestibility across the trial period were analyzed using the GLM repeated measures procedure. When Mauchly’s test indicated that the assumption of sphericity had been violated, the degrees of freedom were corrected using Greenhouse–Geisser estimates if the estimate was lower than 0.75, or the Huynh–Feld estimate if the estimate was greater than 0.75 [27]. The effect sizes for main effects and interactions were determined by partial eta squared (η^2^) values. Partial eta squared (η^2^) values were classified as small (0.01 to 0.059), moderate (0.06 to 0.137) and large (>0.137). 

Differences in intake behavior between second and greater than or equal to third lactating dairy cows were analyzed using an independent samples *t*-test.

The effects of intake behavior on DMI and on the four measured variables (nutrient digestibility of OM, CP, NDF and ADF) were statistically tested using the linear mixed model (LMM) procedure. Analyses were carried out on the individual animal as the observational unit. Akaike’s information criterion and relative standard error (RSE) were, respectively, used for model evaluation and to find the best-fit model. Correlations, calculated with Pearson correlation coefficient, between dependent variables were low (R < 0.80; [28]), indicating that multicollinearity was not a confounding factor in the analysis. The final model to test the effect of intake behavior on DMI included the fixed effects of milk yield, meal frequency, feeding time, meal size, feeding rate and a random intercept for the cow. Meal duration (min/meal) was used to calculate feeding time (min/d) and feeding rate (g/min) and was therefore excluded. There were no significant two-way interaction terms between the fixed effects. The final model to test the effect of intake behavior on nutrient digestibility of OM, CP, NDF and ADF included the fixed effects of week of lactation, DMI (kg/day), meal frequency (meals/d), feeding time (min/d), meal size (kg/meal), feeding rate (g/min) and a random intercept for the cow. There were no significant two-way interaction terms between the fixed effects. Meal duration (min/meal) was used to calculate feeding time (min/d) and feeding rate (g/min) and was therefore excluded from both calculations. 

Cows were retrospectively grouped in quartiles based on average DMI between week 2 and 15 of lactation. The use of quartiles in the formation of groups has the advantage of providing greater differentiation between cows having high differences in DMI. The three groups were created based on the mean DMI of the cows during the trial period. The first group was the lower quartile, consisting of the cows with the lowest mean DMI (<18.99 kg DMI/d, n = 7) between week 2 and 15 of lactation. The second and third groups consisted of the intermediate (between 19.00 and 20.93 kg DMI/d, n = 14) and upper (>20.94 kg DMI/d, n = 7) quartiles, respectively. Differences in intake behavior and nutrient digestibility were analyzed using ANOVA, with quartiles as fixed factor. The individual quartile comparisons were performed using post hoc pair-wise comparisons, with the Sidak correction applied.

## 3. Results

### 3.1. Interactions between Intake Behaviour and DMI during Early Lactation

**Figure 2:**
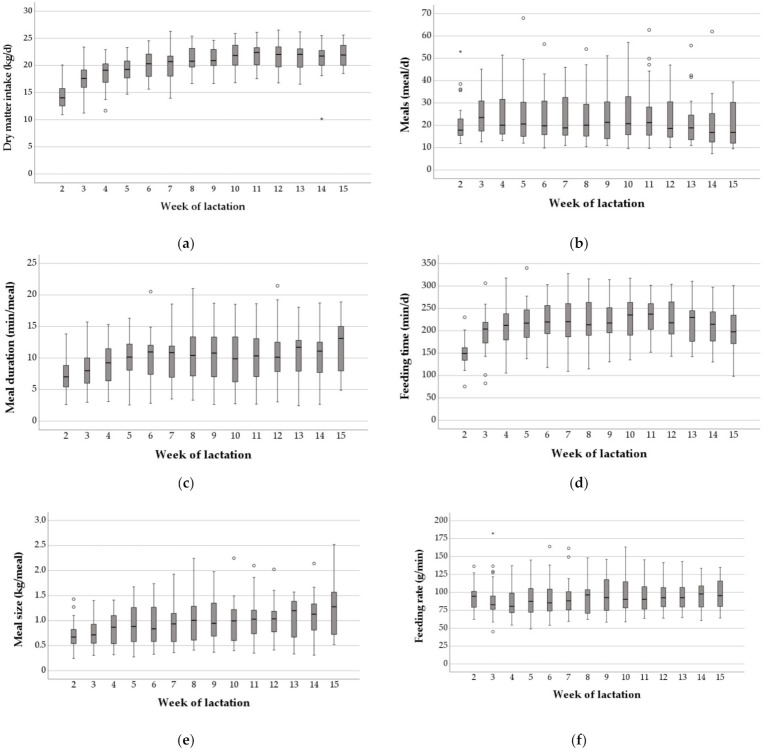
Dry matter intake (kg/day) (**a**), meal frequency (meals/d) (**b**), meal duration (min/meal) (**c**), feeding time (min/d) (**d**), meal size (kg/meal) (**e**) and feeding rate (g/min) (**f**), measured for all 28 cows between week 2 and 15 postpartum (p.p.). In each subfigure, the boxplots highlight, respectively, the median and upper and lower quartiles of each week. Small circles = outliers (1.5 × interquartile range (IQR)); asterisks = extreme values (3 × IQR).

The trends of intake behavior parameters and feed intake over the course of the study are shown in Figure 2. DMI significantly increased with a linear (*p* < 0.001; η^2^ = 0.75) and quadratic (*p* < 0.001; η^2^ = 0.677) trend from a mean value of 14.3 ± 2.3 kg DMI per day in week 2 up to 22.1 ± 2.3 kg in week 11 and 21.57 ± 2.3 kg in week 15 (Figure 2a). Mean CV of DMI ranged between 0.09 (week 9) and 0.17 (week 3). Daily meal frequency remained at the same level of 23.6 ± 11.5 meals over the course of study (*p* > 0.005; η^2^ = 0.122; Figure 2b). Mean CV of daily meal frequency was lowest in week 3 (0.39) and highest in week 14 (0.58). Meal duration had a mean of 9.9 ± 4.0 min/meal and constantly increased with a linear (*p* < 0.001; η^2^ = 0.572) and quadratic (*p* < 0.001; η^2^ = 0.223) trend from 7.4 min/meal in week 2 p.p. to 11.9 min/meal in week 11 p.p. (Figure 2c). CV values ranged from 0.35 (week 14) to 0.45 (week 10). Feeding time per day increased significantly (*p* < 0.001; η^2^ = 0.298) with a linear (*p* < 0.05; η^2^ = 0.385) and quadratic (*p* < 0.001; η^2^ = 0.606) trend from a mean value of 151.2 ± 31.6 min per day in week 2 up to 228.9 ± 48.8 min per day in week 10, and constantly decreased to a value of 209.1 ± 49.8 min in week 15 p.p. (Figure 2d). CV values of total feeding time per day ranged between 0.19 (week 11) and 0.24 (week 7). The meal size had a mean of 0.96 ± 0.4 kg/meal and constantly increased with a linear (*p* < 0.001; η^2^ = 0.602) trend between week 2 and 15 of lactation from 0.7 ± 0.3 kg/meal to 1.3 ± 0.6 kg/meal, respectively (*p* < 0.001; η^2^ = 0.602; Figure 2e), with CV values between 0.35 (week 13) and 0.46 (weeks 8 and 15). The feeding rate remained at the same level of 93.41 ± 22.7 g per min up to week 15 p.p. (*p* > 0.005; η^2^ = 0.122; Figure 2f) and CV ranged between 0.21 (week 13) and 0.30 (week 3). 

The LMM showed significant positive effects of milk yield, meal frequency, feeding time, feeding rate and a comparable high effect of meal size on daily DMI of dairy cows (Table 3).

Table 4 illustrates the mean, SD and CV of DMI and variables of intake behavior for second and greater than or equal to third lactating dairy cows. Mean DMI and SD of meal frequency were significant lower in second lactating cow (*p* = 0.043; *p* = 0.021). No other significant differences were detected between parity groups.

**Figure 3:**
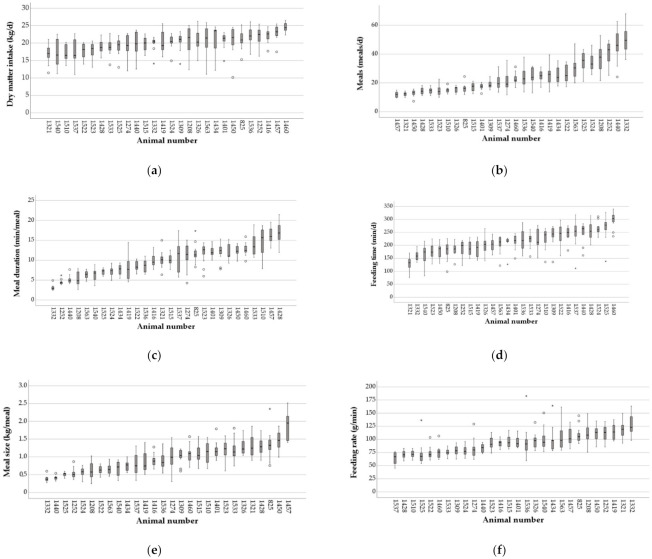
Dry matter intake (kg/day) (**a**), meal frequency (meals/d) (**b**), meal duration (min/meal) (**c**), feeding time (min/d) (**d**), meal size (kg/meal) (**e**) and feeding rate (g/min) (**f**), measured for individual animals (n = 28) between week 2 and 15 postpartum (p.p.). In each subfigure, the boxplots highlight, respectively, the median and upper and lower quartiles of each week. Small circles = outliers (1.5 × interquartile range (IQR)); asterisks = extreme values (3 × IQR).

In addition to the high variation in intake behavior between dairy cows at the same stage of lactation, the extent of variation in intake behavior during early lactation within individual animals was also great (Figure 3). Intra-individual CVs of DMI ranged between 0.05 and 0.25, CVs of meal frequency ranged between 0.11 and 0.36. For meal duration and feeding time, CVs ranged between 0.10 and 0.36 and between 0.08 and 0.26, respectively. The widest range in CVs was detected for meal size (0.1–0.42). Intra-individual CVs for feeding rate ranged between 0.07 and 0.30.

When comparing mean and CV values of intake behavior parameters of cows with mean low, medium and high DMI, no significant differences could be detected between the quartiles (Table 5).

### 3.2. Interactions between Intake Behavior and Nutrient Digestibility during Early Lactation

The highest mean value regarding the nutrient digestibility of the diet was found for OM (728.8 ± 19.8 g/kg), followed by CP, NDF and ADF with 625.4 ± 50.7, 585.1 ± 43.5 and 571.9 ± 34.3 g/kg, respectively. Over the course of the study, mean CV was highest for CP with a value of 0.08 (0.06–0.10) followed by NDF, ADF and OM with 0.07 (0.05–0.10), 0.06 (0.05–0.07) and 0.03 (0.02–0.03), respectively.

The GLM repeated measures procedure reported significant differences between week of lactation for CP (*p* = 0.032; η^2^ = 0.164) and NDF digestibility (*p* = 0.00; η^2^ = 0.427) with a quadratic trend over the time for CP (*p* = 0.041; η^2^ = 0.212) and NDF (*p* = 0.039; η^2^ = 0.215). CP digestibility decreased from 653.4 ± 46.9 g/kg in week 2 to 618.6 ± 60.9 g/kg in week 9, and increased again up to 634.1 ± 48.8 g/kg in week 15. NDF digestibility decreased from 607.4 ± 54.0 g/kg in week 2 to 577.0 ± 36.4 g/kg in week 10 and increased again up to 58.5.4 ± 49.3 g/kg in week 15. No significant differences between the weeks of lactation for OM and ADF digestibility were detected. Variation in OMD within individual animals during early lactation was comparably low with CVs between 0.02 and 0.04. CVs of CPD ranged between 0.04 and 0.11 between animals. CVs of NDFD and ADFD ranged between 0.03 and 0.11, and between 0.03 and 0.08, respectively.

The effects of week of lactation, dry matter intake and intake behavior on nutrient digestibility are shown in Table 6. Significant fixed effects (*p* < 0.05) of DMI were found for nutrient digestibility of OM and CP. Week of lactation had a significant effect on CP and ADFD (*p* < 0.05). Variables of intake behavior had no significant effect on nutrient digestibility. Examination of the components of variance showed that the intercepts significantly varied across cows to the amounts of 50.4, 328.1, 281.1 and 271.4 for OMD, CPD, NDFD and ADFD, respectively. Allowing for all other effects in the model, parameter estimates from this model predicted that nutrient digestibility of OM and CP decreased, respectively, by 1.9 (SE 0.68) and 5.2 (SE 1.7) g/kg DM per kilogram of increase in DMI.

Significant differences could be detected in average and CV values of nutrient digestibility between animals with low mean and high mean feed intake, based on average DMI between week 2 and 15 of lactation of the cows during the trial period, for mean OMD, mean CPD, mean ADF and CV of NDF (Table 7). Mean OMD and CPD in early lactation were significantly higher for animals with mean comparatively low DMI. Mean ADFD and CV of NDFD in early lactation were significantly higher for animals with an average higher DMI.

## 4. Discussion

Considerable variation was observed between individual animals and within animals over time when examining feed intake, intake behavior and nutrient digestibility. To be able to attribute the variation in the measured parameters to the differences between individual animals, possible confounding factors by breed, feed or environment were reduced to a minimum.

### 4.1. Intake Behavior and DMI

The mean values of dry matter intake and daily feeding time obtained in the current study were within the range, but at the lower end, of results which have been reported in other studies [29,30,31]. In the present study, daily DMI increased from 14.3 DMI per day in week 2 up to 22.1 in week 11. A similar increase in DMI during early lactation was described by Azizi et al. [31] and Park et al. [32]. In this context, the latter determined a parallel increase in ruminal capacity as a percentage of body weight of 31.2% during the first 90 days of lactation. DeVries et al. [33] observed an increase in daily mealtime and meal duration from period 1 (35 ± 16 DIM) to period 2 (57 ± 16 DIM) as well, but no changes between period 2 and 3 (94 ± 16 DIM). In contrast, Friggens et al. [34] found no significant effect of the stage of lactation on meal duration and meal size, and likewise, no significant effect of the stage of lactation on meal frequency. However, mean values of meal frequency, meal duration, meal size and feeding rate in the current study were different to the results of other studies [31,35]. A possible explanation for the deviating values may be due to differences in the definition of meal criteria (meals/day). Miron et al. [35], who described an average of 14 meals per day and a meal duration of 15.9 min/meal, defined a meal as a visit to a trough that lasted at least 1 min and eating at least 0.2 kg of TMR. Based on a method developed by Tolkamp et al. [36] and DeVries et al. [33], Azizi et al. [31] calculated a meal criterion of 28.5 min on average. Variation in the results can be also attributed to differences in methodology (e.g., experimental procedures, diet composition, feeding level). Dado and Allen [30] defined a minimum of 7.5 min between events to define two eating periods. They reported by early lactating dairy cows (63 DIM) an average of 24.8 kg DM per day, 10.8 eating bouts per day (meal frequency) with a bout length of 31.1 min (meal duration), a daily feeding time of 314 min and a meal size of 2.5 kg. Because even the smallest amounts of ingested feed are digested, the definition of meal criteria should be examined more closely, especially in studies which focus on digestibility research.

DMI, meal duration, feeding time and meal size increased over the course of study. In contrast, daily meal frequency and feeding rate remained at the same level during study. The average feeding rate in the present study (93.4 g of DM/min) was lower than reported by others; for example, [37] 120.0 g of DM/min, but in agreement with the results shown by Beauchemin et al. [38] (90.0 g of DM/min), even though in that study the cows were housed in individual tie-stalls without competition between cows. In the current study, cows were housed together with non-experimental cows in a free-stall barn with an animal feeding place ratio of 2:1 and the potential for aggressive interactions and displacements from the feed bunk by other cows existed and, in contrast to Beauchemin et al. [38], no primiparous cows were used in the present study. Albright [7] described, that cows tend to consume more feed at a faster rate when they are fed in groups than when fed separately. In the present study, DMI was significantly influenced by variables of intake behavior. Therefore, intake behavior should be considered when developing strategies to increase feed intake. Grant and Albright [10] concluded that management factors, such as grouping strategy, feeding system design and apparatus, composition and physical characteristics of the feed being consumed, as well as social hierarchy and competition for food and water, are the main factors influencing the intake behavior of cattle, especially for cows with a lower standing in the hierarchy.

All parameters of intake behavior showed a substantial variation between dairy cows at the same stage of lactation and within individual dairy cows during the early lactation. An explanation for the variation between dairy cows at the same stage of lactation may be the different milk yields of the 28 cows. Dado and Allen [30] indicated that cows with higher milk yields achieved greater DMI by increasing meal size and decreasing eating time. Azizi et al. [31] also reported 20% less eating time and 28% more DMI in high-yielding dairy cows in comparison to below average yielding cows. Furthermore, higher correlations between intake behavior characteristics and dry matter intake (DMI) within milk yield groups than across all cows were reported [30,34]. Factors which potentially affect the frequency, size and rate of meals could be social interaction between cows [39], feed access [40], palatability and moisture content, as well as the possibility to sort feed, cow health and environmental temperature [39]. No differences, expect a significant lower DMI in second lactating dairy cows, were detected between cows in different parities. Between the categories of low, medium or high dry matter intake, no significant differences in intake behavior parameters and variation were detected, but cows with a higher DMI seemed to be more consistent in intake behavior, except the feeding rate. The high within- and between-cow variability in the current study and in other studies [31,33] indicated that different cows pursue different strategies for nutrient supply. Therefore, it seems even more important to create suitable conditions for the different eating habits to enable high feed intake for all individual cows. Thus, to allow all dairy cows to pursue their own feeding strategy and hence maximize feed intake, the management of young cows should unstintingly focus on designing cow’s individual conditions of feed intake. Especially strategies that avoid sorting of feed in heifers at young age, as well as ensuring a balanced nutrient supply via, e.g., TMR, should be considered [41]. However, also ensuring sufficient space and reducing stress by, e.g., avoiding overcrowding, can contribute to a more even feed intake [42]. The assumption of an almost uniform feed intake at the same stage of lactation is misleading and delusive [43].

### 4.2. Intake Behavior and Nutrient Digestibility

Besides feed intake, nutrient digestibility plays an important role for the available nutrient and energy in dairy cattle [9]. The mean between-cow CV in OM digestibility during the trial period was 0.03. In a recent meta-analysis by Guinguina et al. [44] and in the meta-analysis by Cabezas-Garcia et al. [45], even lower values of 0.014 and 0.013 for between-cow CV in OMD were reported. The results demonstrate that there is little variance among early lactating dairy cows in their ability to digest organic matter of a given diet. In contrast, the variation in digestibility of CP, NDF and ADF with CV values up to 0.08 between cows at the same stage of lactation was at a higher level. Together with the high intra-individual CV values up to 0.11 of CPD, NDFD and ADFD, the variation challenges the traditional approach of generalizing a uniform feed-digestibility value. By using iNDF_240_ instead of tabulated digestibility coefficients, it is possible to identify nutrient digestibility at animal level, and thus the level of nutrients available to individual animals, rather than only the feed level of total digestible nutrients (TDN). Therefore, digestibility assessed by iNDF_240_ from a random sample of fecal samples could be used to evaluate the digestibility of the diet, for example, after a change of the diet. Further research is needed to ascertain a robust sample size for determining digestibility within a herd.

The influence of intake behavior on nutrient digestibility is well described for beef cattle in conjunction with feed efficiency. Robinson and Oddy [46] found phenotypic correlations between feed efficiency and the time spent eating and the number of visits to the feeder of 0.64 and 0.51, respectively. Similar results were reported by Golden et al. [14] and Green et al. [47], who found that efficient cattle ate less and spent less time feeding then inefficient cattle given the same production level. In contrast, research on the influence of intake behavior on nutrient digestibility in dairy cows is rather rare. The presented results do not show any effect of variables of intake behavior on nutrient digestibility but an effect of week of lactation on CPD and ADFD and an effect of dry matter intake on OMD and CPD.

The amount of dry matter consumed and the passage rate are key drivers for the level of digestion, absorption and utilization of feed in the animal. Changes in one of the key drivers lead to changes in the other two [48]. Several researchers have shown that increased intake results in increased passage rates, which is associated with incomplete feed digestion and therefore decreased digestibility [49,50,51]. Colucci et al. [51,52] reported a close relationship (R^2^ = 0.86) between the decrease in ruminal retention time and depression in digestibility in sheep and cows fed different diets. The effect of higher passage rate is especially distinct when the diet contains a high percentage of fiber. With increasing DMI and an increasing passage rate, fiber cannot be sufficiently digested [9]. The same happens in the first weeks after calving, when DMI increases daily. In the present study, DMI increased up to week 11 p.p. Park et al. [32] also reported a rapid increase in dry matter intake from 2.7 to 4.3% of body weight between day 6 and 34 postpartum and then a slower increase up to day 81. Parallel to the increase in dry matter intake, a strong increase in ruminal fill was also observed between days 48 and 62 p.p. [32]. Stafford [53] found a decrease in ruminal motility after day 60 p.p., which could reduce the digesta volume flowing from the rumen, increase the ruminal fill, decrease the passage rate and result in increasing digestibility [32]. Furthermore, the model predicted a positive influence of meal duration on OM and ADF to the amounts of 1.57 and 2.91 g/kg DM per extra minute of eating time per meal. A possible explanation for the positive influence of meal duration may be the increase in salivary secretion, which reduces the size of feed particles [54] and increases rumen fermentation and nutrient digestibility [55].

Significant differences were detected in mean and inter-individual CV values of nutrient digestibility between animals with a low and a high dry matter intake for OMD, CPD, ADF and CV of NDF. Mean OMD and CPD in early lactation was significantly higher for animals with a lower DMI. Mean ADFD and intra-individual CV of NDFD were significantly higher for animals having on average higher DMI during early lactation. Furthermore, the results indicate differences in fiber digestibility between animals, and challenge the assumption of uniform fiber digestibility for all animals fed the same diet.

## 5. Conclusions

The results of this study show a large variation in intake behavior, feed intake and digestibility as well as interactions between these parameters in early lactating dairy cows. The greater the variation in intake behavior and DMI, the greater the variation in nutrient supply and the proportion of animals that are not adequately supplied with energy and nutrients. Because feed intake and feeding behavior appear to be subject to strong individual influence, it is necessary to consider the interaction at the individual animal level rather than inferring potential relationships via averaged group values in order to reduce the individual animal deficits, especially at the beginning of lactation. The described variation in feed intake behavior shows that animals of the same breed, at the same stage of lactation and in the same environment follow different strategies for nutrient and energy intake. The individuality of the feed intake behavior conflicts with herd-based efforts of predicting feed intake. To achieve a sustainable increase in feed intake, and hence nutrient availability, of individual animals, and thus of the herd in the long term, feed conditions must be created that allow for different intake strategies. This includes not only sufficient space at and good access to the feed table, but also a balanced diet and good feeding management. Technical developments that directly assess an individual animal’s DMI should be further advanced to enable better future decisions regarding the supply situation of dairy cows. Only by individually increasing the availability or adjusting the output for each animal can long-term improvement in the health status of early lactating cows be achieved.

## Figures and Tables

**Table 1:** Parity and mean daily milk yield (kg/d) of the trial cows between the 2nd and 15th week of lactation.

Animal	Parity	Mean Daily Milk Yield (kg/d)
825	8	50.3
1208	5	40.5
1252	4	49.0
1274	4	47.0
1309	4	48.2
1321	4	44.9
1326	4	51.4
1332	4	42.2
1401	3	49.4
1416	3	46.6
1419	3	46.5
1428	3	45.4
1434	3	34.4
1440	3	51.6
1450	2	43.5
1457	2	55.5
1460	2	51.4
1510	2	41.3
1515	2	47.4
1522	2	43.5
1523	2	38.5
1524	2	44.0
1525	2	52.1
1533	2	41.7
1536	2	44.6
1537	2	40.4
1540	2	35.7
1563	2	50.0

**Table 2:** Ingredient, chemical composition and energy content of the total mixed ration.

Diet Composition (g/kg DM) ^1^	Mean	SD
Beet pressed pulp silage	188.3	
Grass silage	97.0	
Grass hay	74.7	
Maize silage	259.6	
Concentrate	380.4	
Chemical Composition (g/kg DM) ^1^		
Dry matter	402.0	13.8
OM	931.4	3.8
CP	157.4	8.1
SP	63.2	7.3
EE	43.0	2.4
aNDFom	355.1	8.8
ADF	219.3	4.9
Lignin	28.3	1.2
iNDF_240_	86.4	4.5
Starch	182.3	14.8
ESC	63.1	3.4
TDN ^2^	732.0	5.0
Energy (MJ/kg DM)		
NE_L_^3^	7.0	0.0

^1^ Diet and chemical composition reported on 105 °C dry matter. Averaged values based on weekly conducted feed analysis; diet was offered as TMR. ADF—acid detergent fiber, expressed inclusive of residual ash; aNDFom—neutral detergent fiber assayed with heat-stable amylase and expressed exclusive of residual ash; CP—crude protein; CV—coefficient of variation calculated as ratio of standard deviation to mean; EE—ether extract; ESC—ethanol-soluble carbohydrates; iNDF_240_—indigestible aNDFom; OM—organic matter; SD—standard deviation; SP—soluble protein. ^2^ TDN (g/kg)— total digestibly nutrient values for TMR samples were calculated from the TDN value using Equations (2)–(5) by [17]. ^3^ NE_L_—net energy for lactation, for TMR samples were calculated from the TDN value using Equations (2)–(3) by [17].

**Table 3:** Influence of milk yield and intake behavior on dry matter intake during early lactation in dairy cows, estimated by the linear mixed model (LMM) procedure (n = 28).

	Daily DMI ^1^ (kg/Day)		
Parameter	b ^2^	SE	*p*-Value
Intercept	−7.60	1.02	<0.01
Milk yield (kg/d)	0.12	0.02	<0.01
Meal frequency (meals/d)	0.11	0.02	<0.01
Meal size (kg DM/meal)	4.89	0.41	<0.01
Feeding time (min/d)	0.05	0.00	<0.01
Feeding rate (g/min)	0.05	0.00	<0.01
AIC ^3^	1322.59		

^1^ Dry matter intake. ^2^ Parameter estimate. ^3^ Akaike’s information criterion.

**Table 4:** Mean, SD and CV values of dry matter intake (DMI) and variable of intake behavior of early lactating dairy cows grouped in second and greater than or equal to third lactating dairy cows.

		Parity		
		2	≥3	*p*-Value
DMI ^1^ (kg/d)	mean	19.9	20.3	0.043
	SD	3.4	3.0	0.375
	CV	0.2	0.1	0.301
Meal frequency (meals/d)	mean	21.6	25.7	0.112
	SD	9.0	13.3	0.021
	CV	0.4	0.5	0.427
Meal duration (min/meal)	mean	10.6	9.4	0.581
	SD	3.7	4.3	0.493
	CV	0.3	0.5	0.934
Feeding time (min/d)	mean	222.5	204.3	0.776
	SD	50.4	46.4	0.056
	CV	0.2	0.2	0.251
Meal size (g/meal)	mean	1.0	0.9	0.753
	SD	0.5	0.4	0.984
	CV	0.5	0.4	0.412
Feeding rate (g/min)	mean	86.9	99.9	0.668
	SD	21.5	22.1	0.476
	CV	0.2	0.2	0.324

^1^ Dry matter intake.

**Table 5:** Mean and CV values of DMI and intake behavior of early lactating dairy cows grouped in quartiles according to mean DMI observed between week 2 and 15 of lactation.

	Lower Quartile (<25%)	Intermediate Quartile	Upper Quartile (>75%)	*p*-Value
Mean DMI ^1^	17.61	20.14	22.30	>0.05
CV DMI ^1^	0.15	0.14	0.10	>0.05
Mean meal frequency	18.24	27.62	21.83	>0.05
CV meal frequency	0.19	0.21	0.19	>0.05
Mean meal duration	11.35	8.67	10.85	>0.05
CV meal duration	0.22	0.20	0.15	>0.05
Mean feeding time	206.61	215.51	216.24	>0.05
CV feeding time	0.18	0.14	0.14	>0.05
Mean meal size	1.00	0.82	1.16	>0.05
CV meal size	0.26	0.23	0.22	>0.05
Mean feeding rate	0.08	0.10	0.10	>0.05
CV feeding rate	0.14	0.16	0.16	>0.05

^1^ Dry matter intake.

**Table 6:** Influence of week of lactation, dry matter intake and variables of intake behavior on digestibility of OM, CP, NDF and ADF during early lactation in dairy cows, estimated by linear mixed model (LMM) procedure (n = 28).

	OMD			CPD			NDFD			ADFD		
Variable	Estimate	SE	*p*-Value	Estimate	SE	*p*-Value	Estimate	SE	*p*-Value	Estimate	SE	*p*-Value
Intercept	754.57	11.56	<0.01	714.56	28.79	<0.01	637.31	25.25	<0.01	600.28	19.10	<0.01
Week of lactation	0.29	0.31	0.365	3.00	0.78	<0.01	0.39	0.68	0.561	−1.75	0.51	<0.01
Daily DMI ^1^ (kg/day)	−1.83	0.68	0.008	−5.15	1.70	0.003	−1.95	1.49	0.194	−1.34	1.15	0.244
Meal frequency (meals/day)	−0.06	0.22	0.787	0.11	0.53	0.831	0.09	0.47	0.836	−0.17	0.36	0.650
Meal size (kg/meal)	2.92	6.16	0.636	−1.29	15.37	0.933	0.63	13.56	0.963	2.24	10.44	0.830
Feeding time (min/d)	0.04	0.05	0.368	0.05	0.11	0.659	0.01	0.10	0.898	0.10	0.08	0.213
Feeding rate (kg/min)	−0.03	0.083	0.756	−0.24	−0.21	0.248	−0.02	0.18	0.183	−0.09	0.13	0.520
AIC ^2^	1508.2			2150.9			2055.5			1849.8		

^1^ Dry matter intake. ^2^ Akaike’s information criterion.

**Table 7:** Mean and CV values of nutrient digestibility of early lactating dairy cows grouped in quartiles according to mean DMI observed between week 2 and 15 of lactation.

	Lower Quartile (<25%) (DMI < 18.99 kg DMI/d, n = 7)		Intermediate Quartile (DMI between 19.00 and 20.93 kg DMI/d, n = 14)		Upper Quartile (>75%) (DMI > 20.94 kg DMI/d, n = 7)		*p*-Value
Mean OMD	737.08	^a^	727.17	^b^	726.16	^b^	0.032
CV OMD	0.03		0.02		0.03		>0.05
Mean CPD	638.98	^a^	631.20	^ab^	607.60	^b^	0.032
CV CPD	0.07		0.06		0.09		>0.05
Mean NDFD	600.85		578.72		583.77		>0.05
CV NDFD	0.06	^a^	0.06	^a^	0.08	^b^	0.006
Mean ADFD	574.58	^ab^	561.61	^a^	587.49	^b^	0.021
CV ADFD	0.05		0.06		0.05		>0.05

^a,b^ values with different superscripts within the same line are significantly different.

## Data Availability

Not applicable.

## Abbreviations

ADF	acid detergent fiber
ADFD	digestible acid detergent fiber
aNDFom	neutral detergent fiber assayed with heat-stable amylase and expressed exclusive of residual ash
ANOVA	analysis of variance
CP	crude protein
CPD	digestible crude protein
CV	coefficient of variation
CVAS	Cumberland Valley Analytical Services Inc.
DM	dry matter
DMI	dry matter intake
EE	ether extract
ESC	ethanol-soluble carbohydrates
GLM	generalized linear models
iNDF_240_	240 h in vitro indigestible neutral detergent fiber
LMM	linear mixed model
NDFD	digestible neutral detergent fiber
NEL	net energy for lactation
NIRS	near infrared reflectance spectroscopy
OM	organic matter
OMD	digestible organic matter
RIC	roughage Intake Control
RSE	relative standard error
SD	standard deviation
SP	soluble protein
TDN	total digestibly nutrient
TMR	totally mixed ration
